# Antioxidants from the Brown Alga *Dictyopteris undulata*

**DOI:** 10.3390/molecules23051214

**Published:** 2018-05-18

**Authors:** Momochika Kumagai, Keisuke Nishikawa, Hiroshi Matsuura, Taiki Umezawa, Fuyuhiko Matsuda, Tatsufumi Okino

**Affiliations:** 1 Graduate School of Environmental Science, Hokkaido University, Sapporo 060-0810, Japan; kumagaim@jfrl.or.jp (M.K.); umezawa@ees.hokudai.ac.jp (T.U.); fmatsuda@ees.hokudai.ac.jp (F.M.); 2 Department of Chemistry, Graduate School of Science, Osaka City University, Sumiyoshi-ku, Osaka 558-8585, Japan; knishi@sci.osaka-cu.ac.jp; 3 Japan Food Research Laboratories, Ibaraki, Osaka 567-0085, Japan; 4 National Institute of Technology, Asahikawa College, Asahikawa 071-8142, Japan; matsuura@asahikawa-nct.ac.jp; 5 Faculty of Environmental Earth Science, Hokkaido University, Sapporo 060-0810, Japan

**Keywords:** *Dictyopteris undulata*, antioxidant, sesquiterpene hydroquinone

## Abstract

An investigation of anti-oxidative compounds from the brown alga *Dictyopteris undulata* has led to the isolation and identification of isozonarol, isozonarone, chromazonarol, zonaroic acid and isozonaroic acid. Their structures were identified by comparison of MS and NMR spectra. Full NMR assignment and absolute configuration of isozonaroic acid are described. Isozonarol showed the most potent 1,1-diphenyl-2-picrylhydrazyl (DPPH) radical scavenging activity among the compounds isolated.

## 1. Introduction

Radical species such as active oxygen can promote arteriosclerosis and liver disease by accumulating peroxide in the body. DNA strand breakage and base modification by radical species can cause genetic disorders, carcinogenesis and aging [1,2]. In foods, oxidative denaturation of unsaturated fatty acids causes noxious odor and toxicity [3]. Therefore, antioxidants which prevent such harmful oxidation are important.

As an attempt to search for new antioxidants from natural source materials, we screened extracts of macroalgae and microalgae for 1,1-diphenyl-2-picrylhydrazyl (DPPH) radical scavenging activity. This method is widely used to determine the antioxidant activity [4]. As a screening result, DPPH radical scavenging activities were observed in the lipid-soluble fraction of the red alga *Neorhodomela aculeata* [5] and brown alga *Dictyopteris undulata*. In the present study, we searched for antioxidants from *D. undulata*. and revealed five sesquiterpenoids (**1**–**5**). Since full relative configurations of one of compounds (**5**) has not been determined. We revealed the relative and absolute configuration of isozonaroic acid (**5**) and considered the biosynthetic mechanism of a series of compounds.

## 2. Results and Discussion 

**Figure 1:**
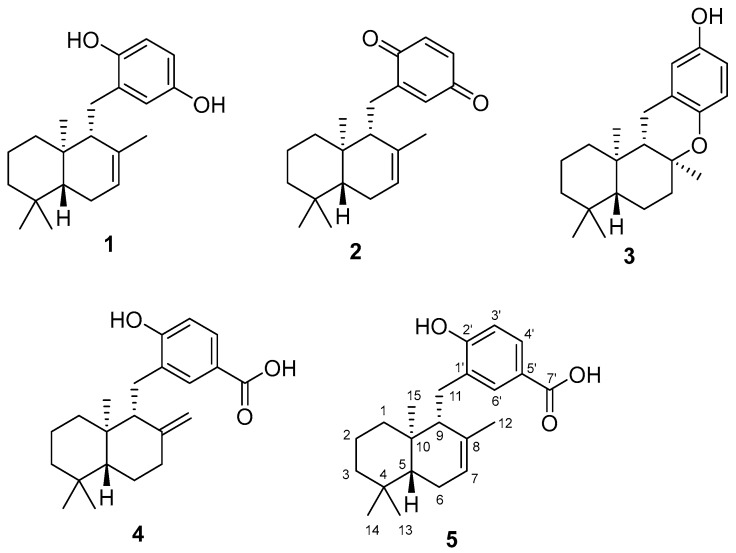
Sesquiterpenoids obtained from *D. undulata* in this study.

In our screening for anti-oxidative substances, we found the methanol extract of *Dictyopteris undulata* had strong DPPH radical scavenge activity. Bioassay-guided fractionation led to isolation of five active compounds: isozonarol (**1**); isozonarone (**2**); chromazonarol (**3**); zonaroic acid (**4**); and isozonaroic acid (**5**). Their structures were identified, as shown in Figure 1, by comparison with the literature data [6,7,8,9,10] and 2D NMR analysis (Supplementary Materials Figures S1–S24). Planer structures and relative configuration of **3** and **4** were determined by analyzing COSY, HSQC, HMBC and NOESY spectra (Supplementary Materials Figures S11 and S18 and Table S1). Absolute configurations of **1**–**4** were reported by chemical synthesis [6,7,11] or chemical degradation [12]. Our experimental values of specific rotation for **1** [α]_D_^18^ +36.6 (*c* 0.075, CHCl_3_), **2** [α]_D_^18^ +115.3 (*c* 0.055, CHCl_3_), **3** [α]_D_^18^ −38.3 (*c* 0.20, CHCl_3_) and **4** [α]_D_^23^ +24.2 (*c* 0.22, CHCl_3_) were similar to the literature values {**1** [α]_D_^22^ +28 (*c* 1.0, CHCl_3_) [6], **2** [α]_D_^21^ +89 (*c* 0.1, MeOH) [6], **3** [α]_D_^20^ −33.5 (*c* 0.37, CHCl_3_) [11] and **4** [α]_D_^27^ +32 (*c* 0.10, CHCl_3_) [13]}. We concluded these structures to be as described in Figure 1. These compounds have been reported from *D. undulata* [8,9,10,11,12,13,14].

**Figure 2:**
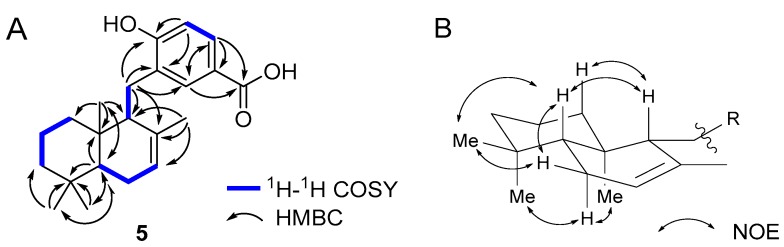
Key COSY, HMBC (**A**) and NOESY (**B**) correlations observed in 2D NMR of isozonaroic acid (**5**).

**Figure 3:**
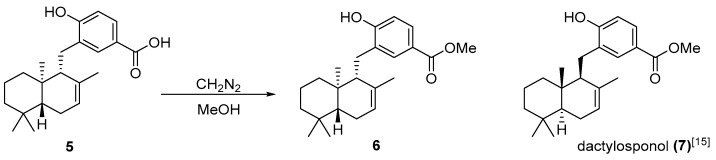
Methyl esterification of isozonaoroic acid (**5**) and structure of dactylosponol (**7**).

**Figure 4:**
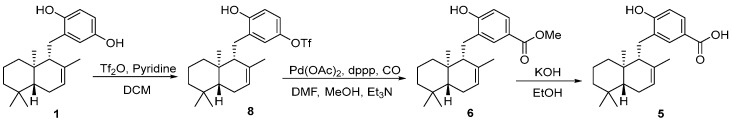
Semisynthesis of isozonaroic acid (**5**) from isozonarol (**1**).

The molecular formula of **5** was determined to be C_22_H_30_O_3_ (*m*/*z* 341.2134, calcd. for C_22_H_29_O_3_, 341.2111 [M − H]^−^) by HR-ESIMS. Compound **5** was suggested to have one more carbon and one oxygen atom than isozonarol (**1**). The ^1^H and ^13^C-NMR spectra of **5** were similar to **1**. However, ^1^H-NMR chemical shift changes were observed (H-4′: δ 6.60 and H-6′: δ 6.74 in **1**, H-4′: δ 7.83 and H-6′: δ 8.03 in **5**). Furthermore, ^13^C-NMR chemical shift (C-7′: δ 171.6) and IR absorption (1678 cm^−1^) indicated that the hydroxy group at C-5′ of **1** was replaced by a carboxylic acid in **5**. Planar structure of **5** was confirmed by COSY and HMBC spectra (Figure 2A). HMBC peaks from H-4′ to C-7′ and H-6′ to C-7′ concluded carboxylation at C-5′. The results indicated that **5** was isozonaroic acid [14]. However, complete assignment of isozonaroic acid (**5**) was not conducted and only the relative configuration between C-9 and C-10 was reported. We assigned all peaks of ^1^H and ^13^C-NMR unambiguously (Supplementary Materials Table S1). All relative configurations in **5** were determined by NOESY correlations (Figure 2B). The absolute configuration of **5** was presumed to be similar to the other analogs isolated from *D. undulata*. To confirm the absolute configuration of **5**, we derivatized to methyl ester **6**, and compared the specific rotation of dactylosponol (**7**) [15] derived from sponge which was enantiomeric to proposed configurations of **6** (Figure 3). Specific rotation of **6** [α]_D_^23^ +12.9 (*c* 0.004, CH_2_Cl_2_) and the literature value of **7** [α]_D_ −14 (*c* 0.05, CH_2_Cl_2_) [15] showed opposite signs, therefore **6** was estimated to be (5*R*, 9*R*, 10*R*). Furthermore, in order to make this result more accurate, we derived isozonaroic acid (**5**) from isozonarol (**1**), which has (5*R*, 9*R*, 10*R*) configurations, and compared the specific rotation of synthetic sample to that of the natural product (Figure 4). Methyl ester **6** was synthesized through triflation of one hydroxy group in **1** followed by palladium-catalyzed alkoxycarbonylation. Subsequent ester hydrolysis afforded the desired **5**. Specific rotations of synthetic **5**, [α]_D_^23^ +48.3 (*c* 0.075, CHCl_3_), and the natural compound, [α]_D_^23^ +48.4 (c 0.055, CHCl_3_), were comparable. Therefore, absolute configurations of **5** are related to the other compounds (**1**–**4**) from *D. undulata*.

The precursor of these sesquiterpenoids is known to be farnesyl diphosphate [9]. Since the stereochemistry of the cyclization reaction product of farnesyl diphosphate is controlled by the folding forms on the substrate surface of the cyclase, it is conceivable that the enantiomer is formed by a single enzyme. Looking at marine natural products, some compounds obtained from the sponge have been reported to have isozonarol-type absolute configurations (5*R*, 9*R*, 10*R*) like hyatellaquinone (from *Heatella intestinalis*) [16,17] and isojaspic acid (from *Cacosponsia* sp.) [18], but those having the opposite absolute configuration such as *ent*-chromazonarol (from *Disidea pallescens*) [19] and *ent*-yahazunol (from *Dysidea* sp.) [20] have also been reported. Given our research and the reports to date, the absolute stereochemistry (5*R*, 10*R*) of all drimane-type sesquiterpenoids isolated from *D. undulata* is formed by a single cyclase that performs this steric regulation.

We tested all five compounds for DPPH radical scavenging activity (Table 1). The intensity of the DPPH radical scavenging activity was highest for isozonarol (**1**), having a hydroquinone structure, and it’s EC_50_ values (71 μM) were comparable to the positive control (α-tocopherol). Hydroquinone structure is known as an active center showing efficient scavenging activity against reactive oxygen species and DPPH radicals [21], and it was thought that DPPH radicals were eliminated by isozonarol (**1**) having hydroquinone structure. Activity was weakened with isozonarone (**2**) and chromazonarol (**3**) without the *para*-hydroquinone structure. In addition, DPPH radical scavenging activity was not observed by replacing one of the hydroxy group with a carboxylic acid (zonaroic acid and isozonaroic acid). These results suggest that the *para*-hydroquinone structure may be important for DPPH radical scavenging activity of these sesquiterpenoids. Although, DPPH assay was foreign to biological systems [22], hydroquinone has a long history of application in cosmetic skin lightning formulations [23]. Recently, anti-inflammatory [24] and neuroprotective effects [25] have been reported for zonarol, which is an analogous substance of isozonarol. Further studies were needed to clarify the biological activities of isolated compounds.

## 3. Materials and Methods

### 3.1. General Procedures

^1^H-NMR and ^13^C-NMR spectra were recorded in CDCl_3_ by using JEOL JNM ECA-600 (JEOL, Tokyo, Japan), JEOL JNM-EX400 (JEOL, Tokyo, Japan). ESI-MS were obtained on a Bruker Daltonics micrOTOF-HS focus spectrometer. Optical rotations were recorded on a HORIBA SEPA-300 polarimeter (Horiba, Kyoto, Japan). IR spectra were measured on a JASCO IR-700 spectrometer (JASCO, Tokyo, Japan).

### 3.2. Plant Material

Algal samples of *Dictyopteris undulata* were collected at Chichi-jima Island and Haha-jima Island, Tokyo, Japan.

### 3.3. Isolation of Compounds

The air-dried alga (40.5 g) was soaked in MeOH (0.5 L ×2). The MeOH solution was concentrated in vacuo, and the residue was partitioned between EtOAc (500 mL) and H_2_O (500 mL). The EtOAc layer was then concentrated to leave a dark green substance (2.5 g). The extract (2.45 g) was fractioned by Si gel CC with a step gradient (hexane and EtOAc) to give thirteen fractions. A part of the third fraction (68.8 mg) which was eluted with hexane-EtOAc (9:1) was subjected to RP-HPLC (Cosmosil 5C18AR-II with MeOH) to give isozonarone (**2**, 2.6 mg). The forth fraction (205.9 mg) was further separated by RP-HPLC (Inertsil-ODS-3, MeCN and H_2_O) to give chromazonarol (**3**, 6.0 mg). The fifth fraction (1023 mg) which was eluted with hexane-EtOAc (4:1) was further separated by Si gel column chromatography with a step gradient (hexane and EtOAc). The fraction (452 mg) eluted with hexane-EtOAc (4:1) was further subjected to NP-HPLC (Develosil 30-5, hexane and 2-propanol) to give isozonarol (**1**, 171 mg). The eighth fraction (191 mg) eluted with hexane-EtOAc (1:1) was separated by RP-HPLC (Cosmosil 5C18AR-II with MeOH and H_2_O) to give zonaroic acid (**4**, 20.0 mg) and isozonaroic acid (**5**, 7.5 mg). The following shows the NMR data for the isolated compounds.

Isozonarol (**1**): ^1^H-NMR (400 MHz, CDCl_3_): δ 0.89 (s, 3H), 0.90 (s, 3H), 0.92 (s, 3H), 1.08–1.31 (m, 3H), 1.42–1.48 (m, 5H), 1.55 (m, 1H), 1.90 (m, 2H), 1.98 (m, 1H), 2.36 (brs, 1H), 2.59 (m, 2H), 5.39 (brs, 1H), 6.50 (dd, *J* = 8.3/2.9 Hz, 1H), 6.60 (d, *J* = 8.6 Hz, 1H), 6.74 (d, *J* = 2.9 Hz, 1H); ^13^C-NMR (100 MHz, CDCl_3_): δ 14.1, 19.1, 22.1, 22.4, 23.9, 26.3, 33.2, 33.4, 37.0, 39.7, 42.4, 50.4, 54.3, 112.7, 115.9, 116.4, 122.2, 131.2, 135.2, 146.8, 149.1; ESI-HR-MS: *m*/*z* 313.2182 [M − H]^−^ (calcd for C_21_H_29_O_2_, 313.2168); [α]_D_^18^ +36.6 (*c* 0.075, CHCl_3_).

Isozonarone (**2**): ^1^H-NMR (400 MHz, CDCl_3_): δ 0.85 (s, 3H), 0.88 (s, 3H), 0.90 (s, 3H), 1.04 (m, 1H), 1.19 (m, 1H), 1.26 (m, 1H), 1.40–1.60 (m, 3H), 1.46 (brs, 3H), 1.78 (m, 1H), 1.90 (m, 1H), 2.01 (m, 1H), 2.20 (m, 1H), 2.39 (m, 1H), 2.55 (m, 1H), 5.43 (m, 1H), 6.67 (m, 1H), 6.72 (dd, *J* = 10.0/2.4 Hz, 1H), 6.78 (d, *J* = 10.0 Hz, 1H ); ^13^C-NMR (100 MHz, CDCl_3_): δ 14.0, 19.0, 22.0, 22.8, 23.9, 25.9, 33.1, 33.3, 36.9, 39.8, 42.2, 50.2, 53.0, 123.3, 132.6, 133.4, 135.9, 136.8, 151.4, 187.1, 187.3; APCI-HR-MS: *m*/*z* 312.2054 [M]^−^ (calcd for C_21_H_29_O_2_, 312.2084); [α]_D_^18^ +115.3 (*c* 0.055, CHCl_3_).

Chromazonarol (**3**): ^1^H-NMR (400 MHz, CDCl_3_): δ 0.84 (s, 3H), 0.88 (s, 3H), 0.90 (s, 3H), 0.95 (m, 1H), 1.02 (m, 1H), 1.14–1.19 (m, 1H), 1.17 (s, 3H), 1.35–1.46 (m, 3H), 1.62–1.74 (m, 5H), 2.03 (m, 2H), 2.56 (m, 1H), 6.55 (d, *J* = 10.3 Hz, 2H), 6.62 (d, *J* = 8.3 Hz, 1H); ^13^C-NMR (100 MHz, CDCl_3_): δ 14.8, 18.5, 19.7, 20.7, 21.6, 22.5, 33.2, 33.4, 36.8, 39.2, 41.1, 41.8, 52.1, 56.1, 76.7, 114.2, 115.8, 117.5, 123.3, 147.2, 148.5; ESI-HR-MS: *m*/*z* 313.2225 [M − H]^−^ (calcd for C_21_H_29_O_2_, 313.2168); [α]_D_^18^ −38.3 (*c* 0.20, CHCl_3_). Detailed ^1^H-NMR and ^13^C-NMR spectra, see Supplementary Materials Table S1.

Zonaroic acid (**4**): ^1^H-NMR (400 MHz, CDCl_3_): δ 0.83 (s, 3H), 0.84 (s, 3H), 0.90 (s, 3H), 1.20 (m, 1H), 1.21 (m, 1H), 1.24 (m, 1H), 1.36–1.42 (m, 2H), 1.52 (m, 1H), 1.61 (m, 1H), 1.76 (m, 1H), 1.88 (m, 1H), 2.05 (m, 1H), 2.26 (m, 1H), 2.38 (m, 1H), 2.78 (m, 2H), 4.67 (s, 1H), 4.81 (s, 1H), 6.77 (d, *J* = 8.3 Hz, 1H), 7.81 (dd, *J* = 8.3/1.9 Hz, 1H), 7.87 (d, *J* = 1.7 Hz, 1H); ^13^C-NMR (100 MHz, CDCl_3_): δ 14.5, 19.4, 21.7, 23.4, 24.4, 33.6, 33.6, 38.1, 39.1, 40.1, 42.1, 55.5, 55.8, 107.6, 115.0, 121.3, 128.6, 129.5, 132.3, 148.7, 158.8, 171.9; ESI-HR-MS: *m*/*z* 341.2103 [M − H]^−^ (calcd for C_22_H_29_O_3_, 341.2111); [α]_D_^23^ +24.2 (*c* 0.22, CHCl_3_). Detailed ^1^H-NMR and ^13^C-NMR spectra, see Supplementary Materials Table S1.

Isozonaroic acid (**5**): ^1^H-NMR (400 MHz, CDCl_3_): δ 0.89 (s, 3H), 0.91 (s, 3H), 0.92 (s, 3H), 1.16 (m, 1H), 1.23 (m, 1H), 1.33 (m, 1H),1.43–1.47 (m, 2H), 1.44 (brs, 3H), 1.57 (m, 1H), 1.90–2.00 (m, 3H), 2.46 (brs, 1H), 2.67 (m, 2H), 5.41 (brs, 1H), 6.78 (d, *J* = 8.3 Hz, 1H), 7.83 (d, *J* = 8.3 Hz, 1H), 8.03 (s, 1H); ^13^C-NMR (100 MHz, CDCl_3_): δ 13.9, 18.9, 22.0, 22.4, 23.8, 26.0, 33.1, 33.2, 36.9, 39.5, 42.1, 50.1, 53.8, 115.2, 121.7, 122.7, 129.4, 130.0, 132.5, 135.0, 158.0, 171.6; ESI-HR-MS: *m*/*z* 341.2134 [M − H]^−^ (calcd for C_22_H_29_O_3_, 341.2111); [α]_D_^23^ +48.4 (*c* 0.055, CHCl_3_); IR (neat) ν_max_ 3378, 2916, 1678, 1599, 1271, 1125, 1093, 1043, 928; Detailed ^1^H-NMR and ^13^C-NMR spectra, see Supplementary Materials Table S1.

### 3.4. Methylation of Isozonaroic Acid *(**5**)*

To a solution of **5** (3 mg, 0.009 mmol) in MeOH (0.1 mL) was added CH_2_N_2_ in Et_2_O (0.24 mL) at 0 °C for 2 h. The mixture was concentrated under reduced pressure and purified by silica gel column chromatography (Hex/EtOAc, 7:3). The following shows the NMR data for compound (**6**).

Isozonaroic acid methyl ester (**6**): ^1^H-NMR (400 MHz, CDCl_3_): δ 0.89 (s), 0.91 (s), 0.92 (s), 1.43 (brs), 2.47 (m), 2.65 (m), 3.86 (s), 5.40 (brs), 6.74 (d), 7.75 (dd), 7.95 (d); ESI-HR-MS: *m*/*z* 355.2286 [M − H]^−^ (calcd for C_23_H_32_O_3_, 355.2273); [α]_D_^23^ +12.9 (*c* 0.004, CH_2_Cl_2_).

### 3.5. Semisynthesis of Isozonaroic Acid from Isozonarol

Triflation of one hydroxy group in isozonarol (**1**) followed by palladium-catalyzed alkoxycarbonylation was conducted [26,27]. Isolated compound **1** (70 mg, 0.22 mmol) was dissolved in dichloromethane (DCM) (5.0 mL). After the solution was cooled to −78 °C, pyridine (70 µL, 0.87 mmol) was added dropwise to the solution. A solution of triflic anhydride (77 µL, 0.47 mmol) in DCM (2.2 mL) was added dropwise to the solution. After addition, the reaction mixture was warmed to room temperature and stirred for 30 min. Then the reaction mixture was partitioned between EtOAc, brine, and 1 M HCl aqueous. The organic layer was washed with H_2_O and brine, dried over anhydrous Na_2_SO_4_, filtered, and concentrated *in vacuo*. Purification by RP-HPLC (Cosmosil 5C18AR-II with MeOH) to give triflate **8** (46 mg, 0.10 mmol, 46%) as white powder.

Triflate **8** (15 mg, 0.034 mmol), Pd(OAc)_2_ (8.4 mg, 0.037 mmol), and dppp (14 mg, 0.034 mmol) were dissolved to the solution (DMF/MeOH/Et_3_N, 5:5:1, 2.0 mL) under the CO atmosphere. After the solution was stirred at 60 °C for overnight, the reaction mixture was partitioned between EtOAc, brine, and 1 M HCl aqueous. The organic layer was washed with H_2_O and brine, dried over anhydrous Na_2_SO_4_, filtered, and concentrated *in vacuo*. Since unreacted compound **8** and methyl ester **6** could not be separated by silica gel CC (hexane/EtOAc, 7:3) and RP-HPLC, the reaction mixture (4.5 mg) was dissolved in EtOH (126 µL) and 63% KOH aqueous (112 µL) and stirred for overnight. Then the reaction mixture was partitioned between EtOAc, brine, and 1 M HCl aqueous. The organic layer was washed with H_2_O and brine, dried over anhydrous Na_2_SO_4_, filtered, and concentrated *in vacuo*. It then underwent purification by RP-HPLC (Cosmosil 5C18AR-II with MeOH/H_2_O, 9:1) to give isozonaroic acid (**5**) (1.5 mg, 0.0044 mmol, 13% in 2 steps) as a colorless solid. ^1^H and ^13^C-NMR chemical shifts of synthetic **5** were identical to those of the natural sample. Specific rotation of synthetic **5** as follows; [α]_D_^23^ +48.3 (*c* 0.075, CHCl_3_).

### 3.6. DPPH Radical Scavenge Assay

Isolated compounds were dissolved in ethanol. This solution (20 µL) was added to 80 µL of Tris-HCl buffer (pH 7.4) on a 96 well plate. DPPH (1,1-diphenyl-2-picrylhydrazyl) was dissolved (0.5 mM) in ethanol and added (100 µL) to start the reaction and incubated at room temperature for 20 min. After incubation, the absorbance was recorded at 570 nm using a micro plate reader. Results were expressed as percentage decrease with respect to control values. The control sample contained solvent (ethanol) in place of the test sample. α-Tocopherol was used as the reference sample. All assays were conducted in triplicate or duplicate.

## 4. Conclusions

Sesquiterpenoids (**1**–**5**) were identified as DPPH radical scavenger from brown alga, *Dictyopteris undulata*. The absolute stereochemistry of isozonaroic acid, which has not been reported, was determined to be (5*R*, 9*R*, 10*R*) by 2D NMR analysis and chemical derivatization. Among the isolated compounds, DPPH radical scavenge activity of isozonarol, having a hydroquinone structure, was the strongest.

## Figures and Tables

**Table 1:** 1,1-diphenyl-2-picrylhydrazyl (DPPH) radical scavenging activities of isolated compounds.

Compound Name	DPPH Radical
	Scavenging Activity
	EC_50_ Value (µM)
Isozonarol (**1**)	71
Isozonarone (**2**)	145
Chromazonarol (**3**)	121
Zonaroic acid (**4**)	>1000
Isozonaroic acid (**5**)	>1000
α-Tocopherol	74

## Supplementary Materials

The following are available online, Figure S1: ^1^H-NMR spectrum of isozonarol (**1**) in CDCl_3_; Figure S2: ^13^C-NMR spectrum of isozonarol (**1**) in CDCl_3_; Figure S3: ^1^H-NMR spectrum of isozonarone (**2**) in CDCl_3_; Figure S4: ^13^C-NMR spectrum of isozonarone (**2**) in CDCl_3_; Figure S5: ^1^H-NMR spectrum of chromazonarol (**3**) in CDCl_3_; Figure S6: ^13^C-NMR spectrum of chromazonarol (**3**) in CDCl_3_; Figure S7: COSY spectrum of chromazonarol (**3**) in CDCl_3_; Figure S8: HSQC spectrum of chromazonarol (**3**) in CDCl_3_; Figure S9: HMBC spectrum of chromazonarol (**3**) in CDCl_3_; Figure S10: NOESY spectrum of chromazonarol (**3**) in CDCl_3_; Figure S11: Key COSY, HMBC (A) and NOESY correlations of chromazonarol (**3**); Figure S12: ^1^H-NMR spectrum of zonaroic acid (**4**) in CDCl_3_; Figure S13: ^13^C-NMR spectrum of zonaroic acid (**4**) in CDCl_3_; Figure S14: COSY spectrum of zonaroic acid (**4**) in CDCl_3_; Figure S15: HSQC spectrum of zonaroic acid (**4**) in CDCl_3_; Figure S16: HMBC spectrum of zonaroic acid (**4**) in CDCl_3_; Figure S17: NOESY spectrum of zonaroic acid (**4**) in CDCl_3_; Figure S18: Key COSY, HMBC (A) and NOESY correlations of zonaroic acid (**4**); Figure S19: ^1^H-NMR spectrum of isozonaroic acid (**5**) in CDCl_3_; Figure S20: ^13^C-NMR spectrum of isozonaroic acid (**5**) in CDCl_3_; Figure S21: COSY spectrum of isozonaroic acid (**5**) CDCl_3_; Figure S22: HSQC spectrum of isozonaroic acid (**5**) in CDCl_3_; Figure S23: HMBC spectrum of isozonaroic acid (**5**) in CDCl_3_; Figure S24: NOESY spectrum of isozonaroic acid (**5**) in CDCl_3_; Table S1: 1H and 13C-NMR spectroscopic data for chromazonarol (**3**), zonaroic acid (**4**) and isozonaroic acid (**5**) in CDCl_3_.
