# Supplementary material for: Antioxidants from the Brown Alga Dictyopteris undulata
Source: Molecules. 2018 May 18;23(5):1214. doi: 10.3390/molecules23051214 (PMC6099395; doi:10.3390/molecules23051214)
Supplement: Supplementary file 1 [file molecules-23-01214-s001.pdf]

Supplementary materials

Communication

Antioxidants from the Brown Alga *Dictyopteris undulata*

Momochika Kumagai [1,2,3], Keisuke Nishikawa [2], Hiroshi Matsuura [4], Taiki Umezawa [1,5], Fuyuhiko Matsuda [1,5] and Tatsufumi Okino [1,5,*]

1 Graduate School of Environmental Science, Hokkaido University, Sapporo 060-0810, Japan; kumagaim@jfrl.or.jp (M.K.); umezawa@ees.hokudai.ac.jp (T.U.); fmatsuda@ees.hokudai.ac.jp (F.M.)
2 Department of Chemistry, Graduate School of Science, Osaka City University, Sumiyoshi-ku, Osaka 558-8585, Japan; knishi@sci.osaka-cu.ac.jp
3 Japan Food Research Laboratories, Ibaraki, Osaka, 567-0085, Japan
4 National Institute of Technology, Asahikawa College, Asahikawa 071-8142, Japan; matsuura@asahikawa-nct.ac.jp
5 Faculty of Environmental Earth Science, Hokkaido University, Sapporo 060-0810, Japan
* Correspondence: okino@ees.hokudai.ac.jp; Tel.: +81-11-706-4519

Contents of Supporting Information

Figure S1: ^{1}H NMR spectrum of isozonarol (**1**) in $CDCl_3$

Figure S2: ^{13}C NMR spectrum of isozonarol (**1**) in $CDCl_3$

Figure S3: ^{1}H NMR spectrum of isozonarone (**2**) in $CDCl_3$

Figure S4: ^{13}C NMR spectrum of isozonarone (**2**) in $CDCl_3$

Figure S5: ^{1}H NMR spectrum of chromazonarol (**3**) in $CDCl_3$

Figure S6: ^{13}C NMR spectrum of chromazonarol (**3**) in $CDCl_3$

Figure S7: COSY spectrum of chromazonarol (**3**) in $CDCl_3$

Figure S8: HSQC spectrum of chromazonarol (**3**) in $CDCl_3$

Figure S9: HMBC spectrum of chromazonarol (**3**) in $CDCl_3$

Figure S10: NOESY spectrum of chromazonarol (**3**) in $CDCl_3$

Figure S11: Key COSY, HMBC (A) and NOESY correlations of chromazonarol (**3**)

Figure S12: ^{1}H NMR spectrum of zonaroic acid (**4**) in $CDCl_3$

Figure S13: ^{13}C NMR spectrum of zonaroic acid (**4**) in $CDCl_3$

Figure S14: COSY spectrum of zonaroic acid (**4**) in $CDCl_3$

Figure S15: HSQC spectrum of zonaroic acid (**4**) in $CDCl_3$

Figure S16: HMBC spectrum of zonaroic acid (**4**) in $CDCl_3$

Figure S17: NOESY spectrum of zonaroic acid (**4**) in $CDCl_3$

Figure S18: Key COSY, HMBC (A) and NOESY correlations of zonaroic acid (**4**)

Figure S19: ^{1}H NMR spectrum of isozonaroic acid (**5**) in $CDCl_3$

Figure S20: ^{13}C NMR spectrum of isozonaroic acid (**5**) in $CDCl_3$

Figure S21: COSY spectrum of isozonaroic acid (**5**) $CDCl_3$

Figure S22: HSQC spectrum of isozonaroic acid (**5**) in $CDCl_3$

Figure S23: HMBC spectrum of isozonaroic acid (**5**) in $CDCl_3$

Figure S24: NOESY spectrum of isozonaroic acid (**5**) in $CDCl_3$

Table S1: ^{1}H and ^{13}C NMR spectroscopic data for chromazonarol (**3**), zonaroic acid (**4**) and isozonaroic acid (**5**) in $CDCl_3$

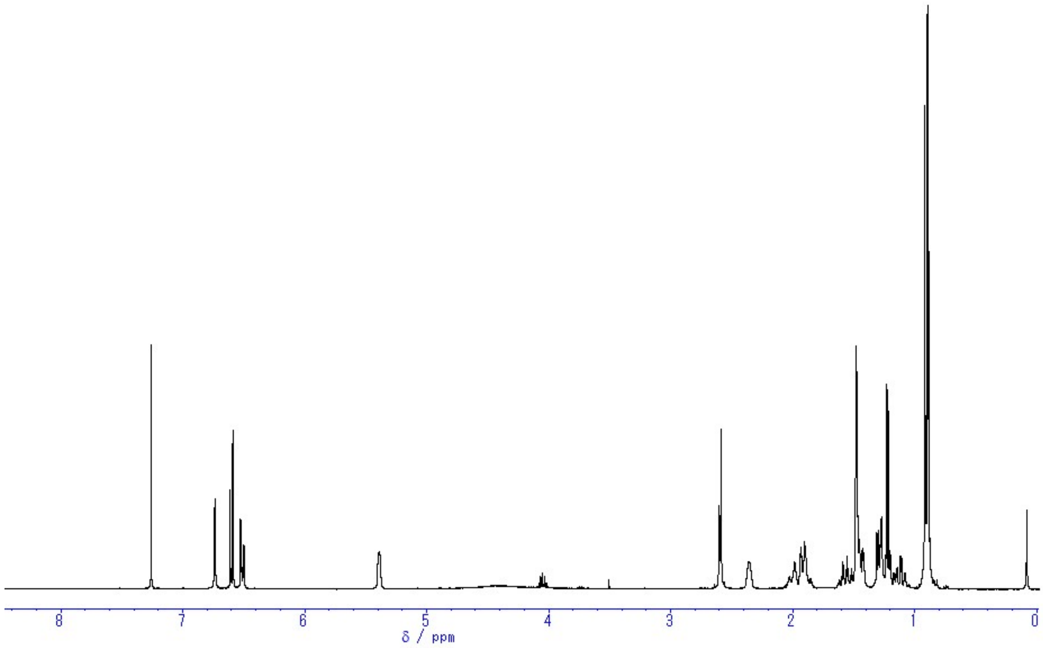

Figure S1: ^{1}H NMR spectrum of isozonarol in $CDCl_3$

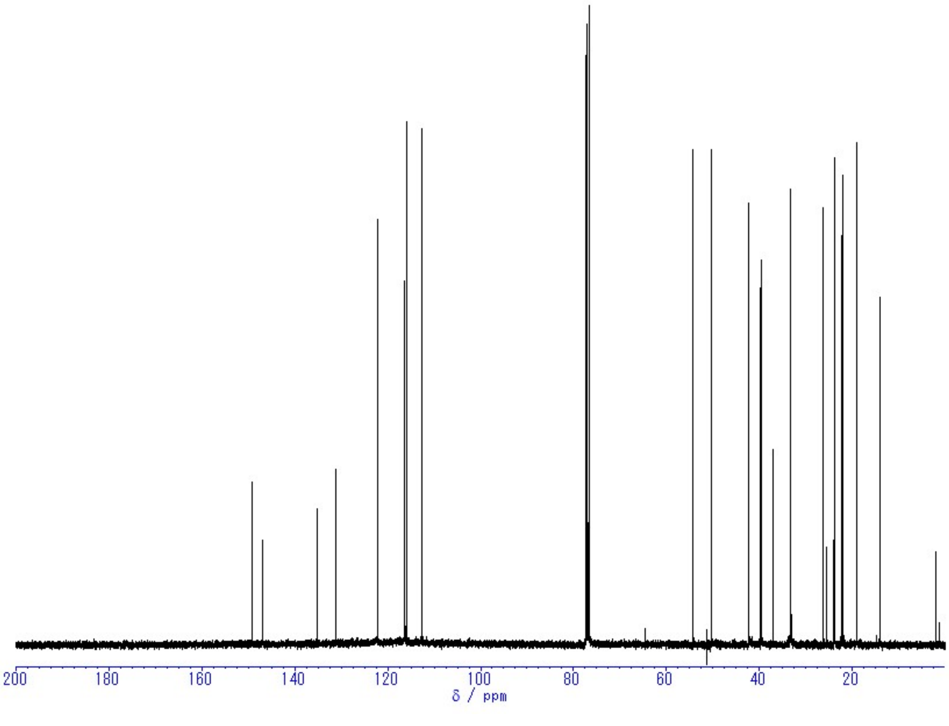

Figure S2: ^{13}C NMR spectrum of isozonarol in $CDCl_3$

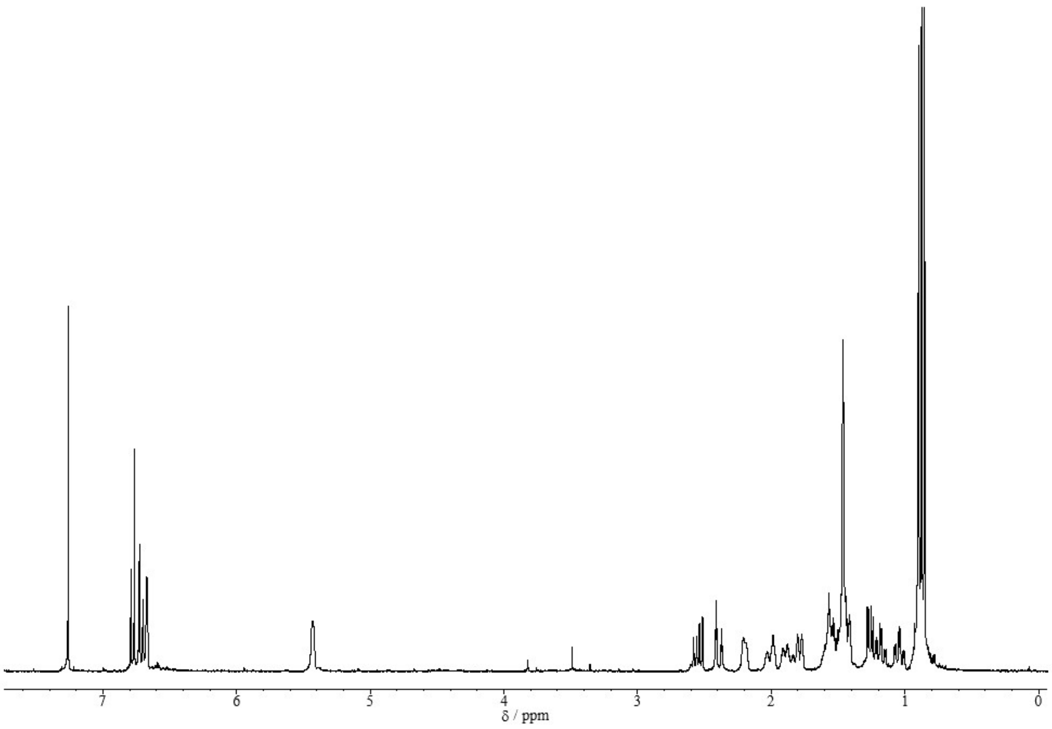

Figure S3: ^{1}H NMR spectrum of isozonarone in $CDCl_3$

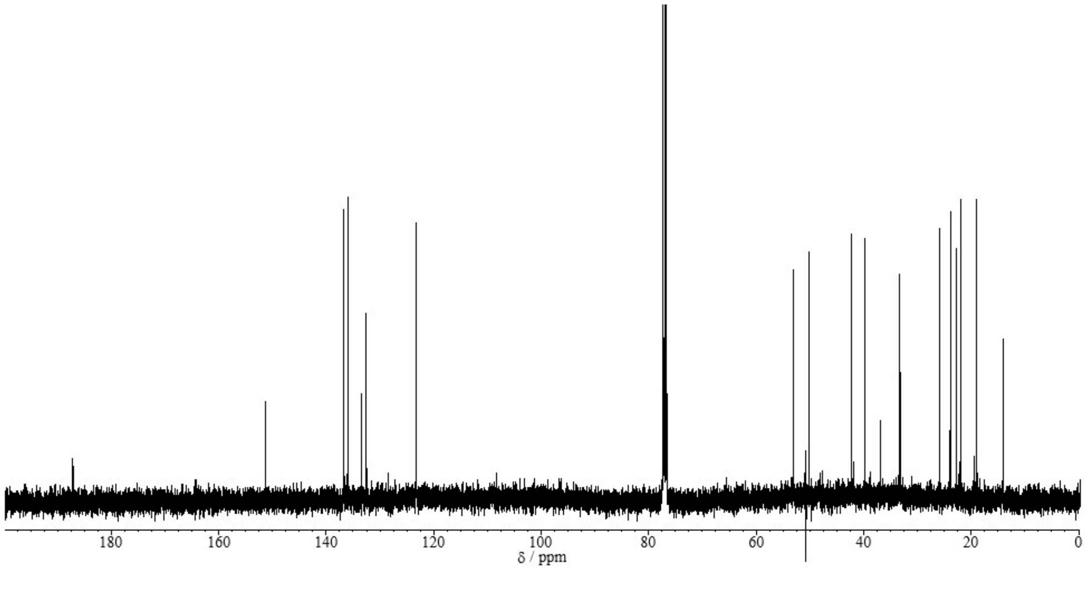

Figure S4: ^{13}C NMR spectrum of isozonarone in $CDCl_3$

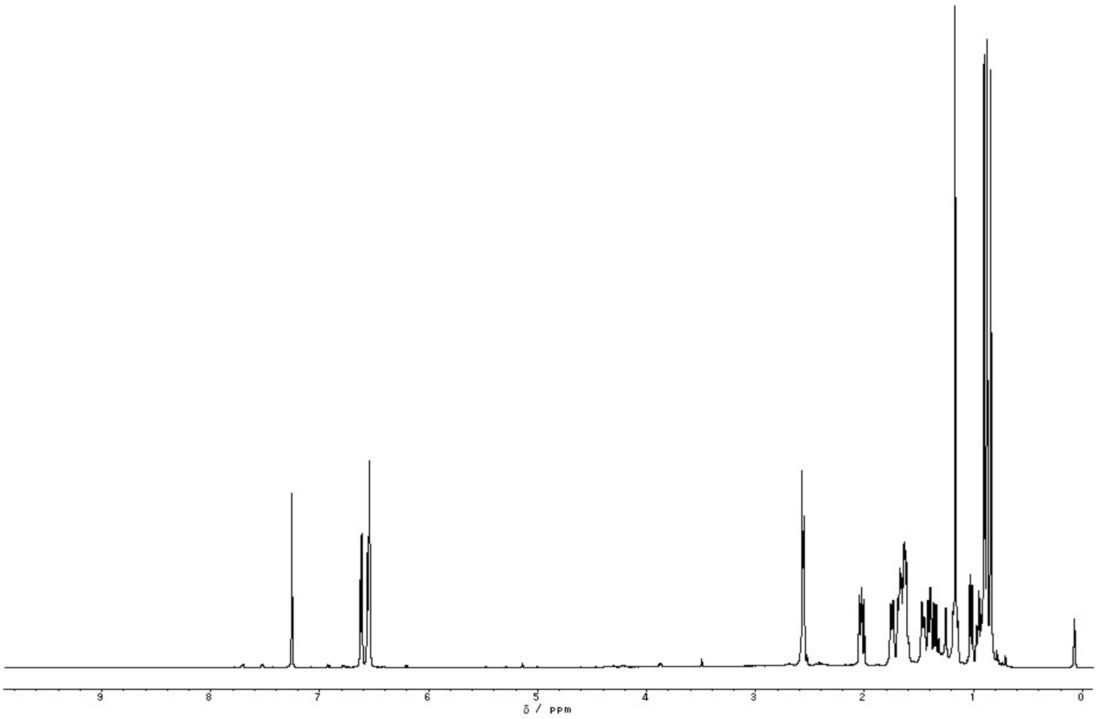

Figure S5: ^{1}H NMR spectrum of chromazonarol (**3**) in $CDCl_3$

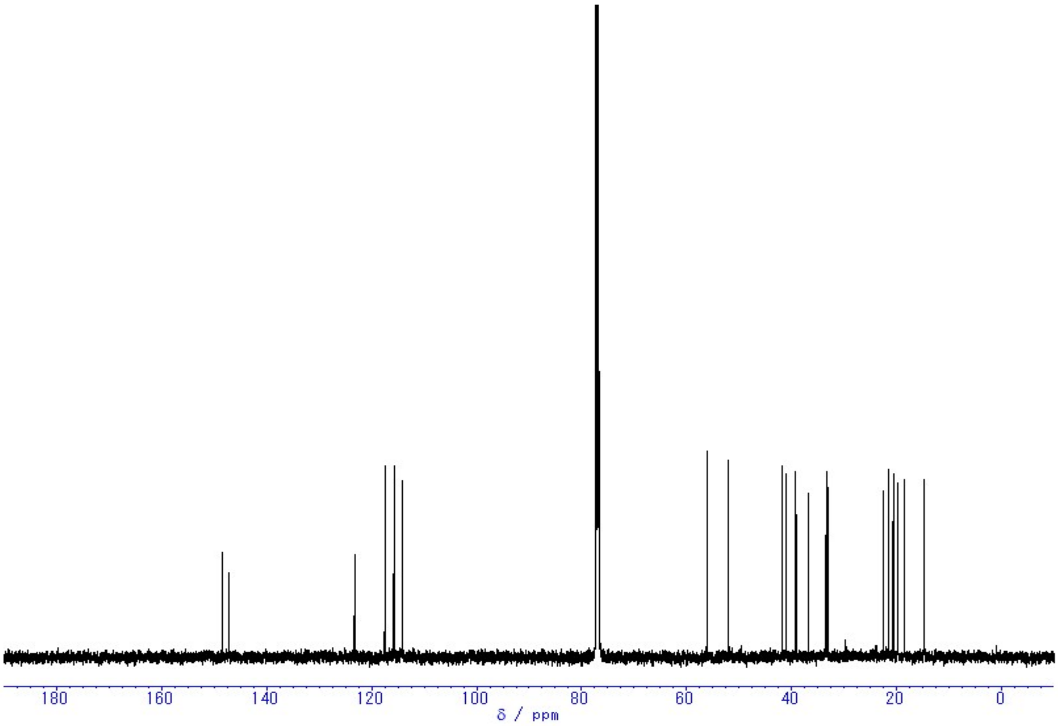

Figure S6: ^{13}C NMR spectrum of chromazonarol (**3**) in $CDCl_3$

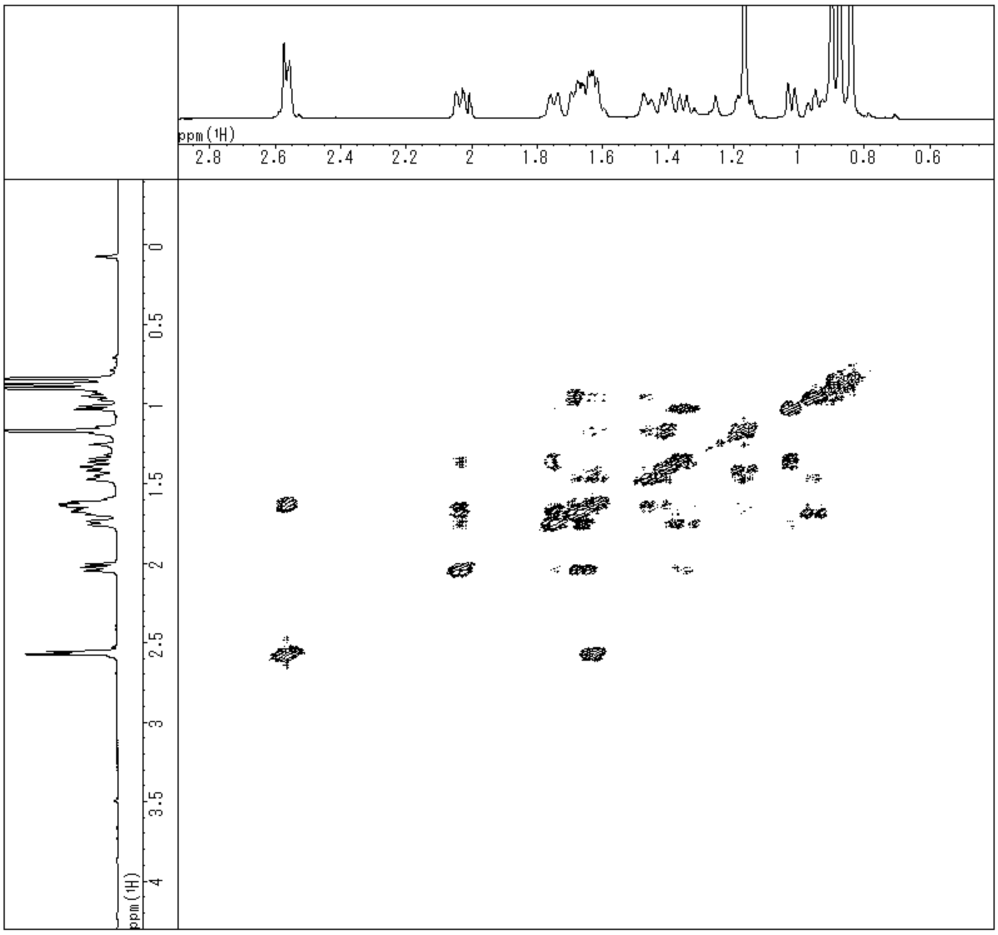

Figure S7: COSY spectrum of chromazonarol (**3**) in $CDCl_3$

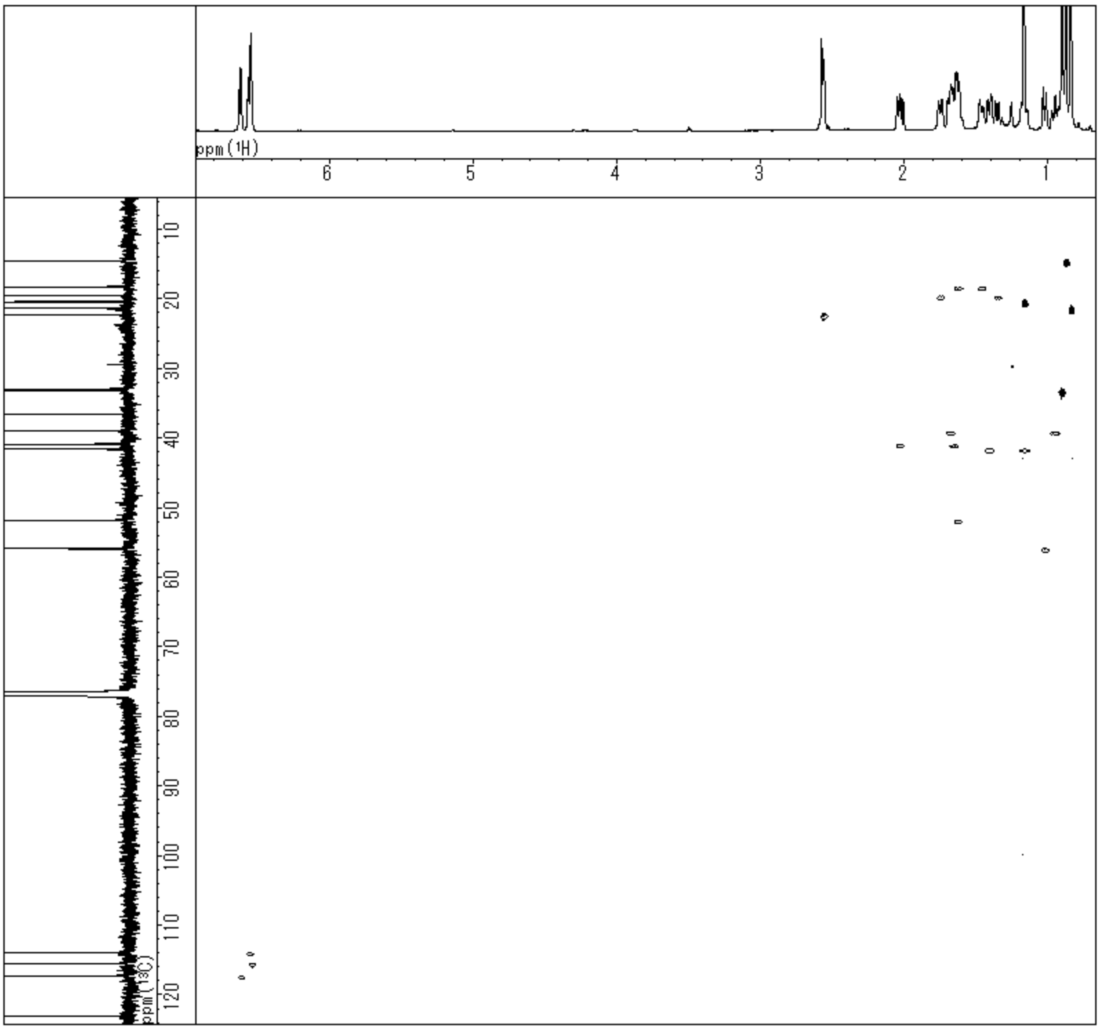

Figure S8: HSQC spectrum of chromazonarol (**3**) in $CDCl_3$

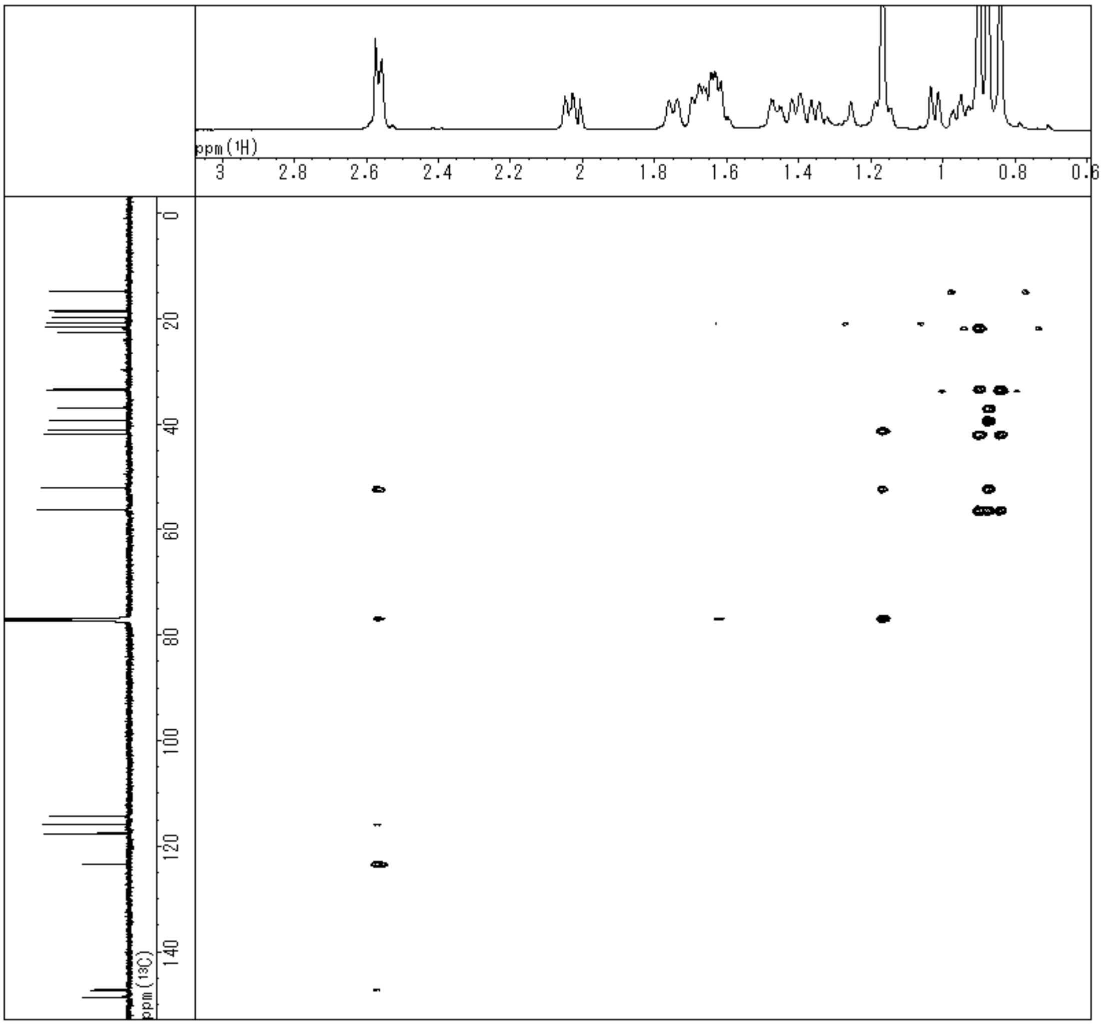

Figure S9: HMBC spectrum of chromazonarol (**3**) in $CDCl_3$

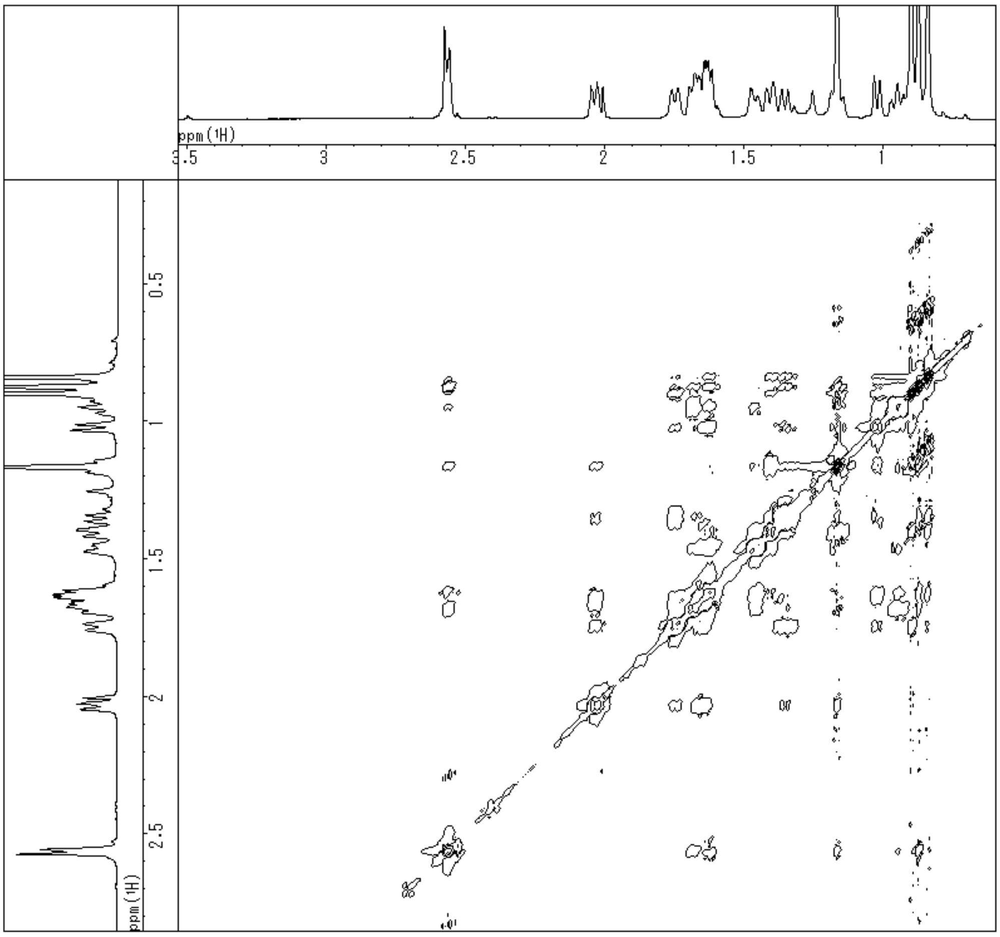

Figure S10: NOESY spectrum of chromazonarol (**3**) in $CDCl_3$

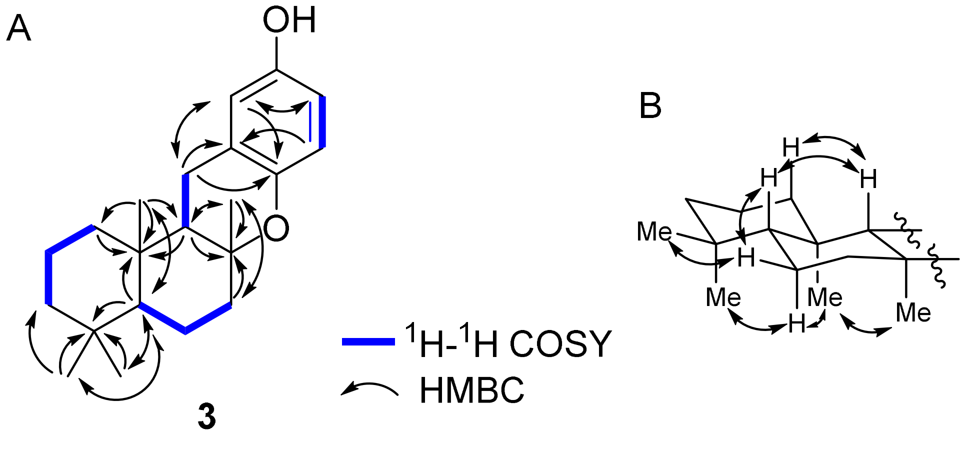

Figure S11: Key COSY, HMBC (A) and NOESY (B) correlations of chromazonarol (**3**)

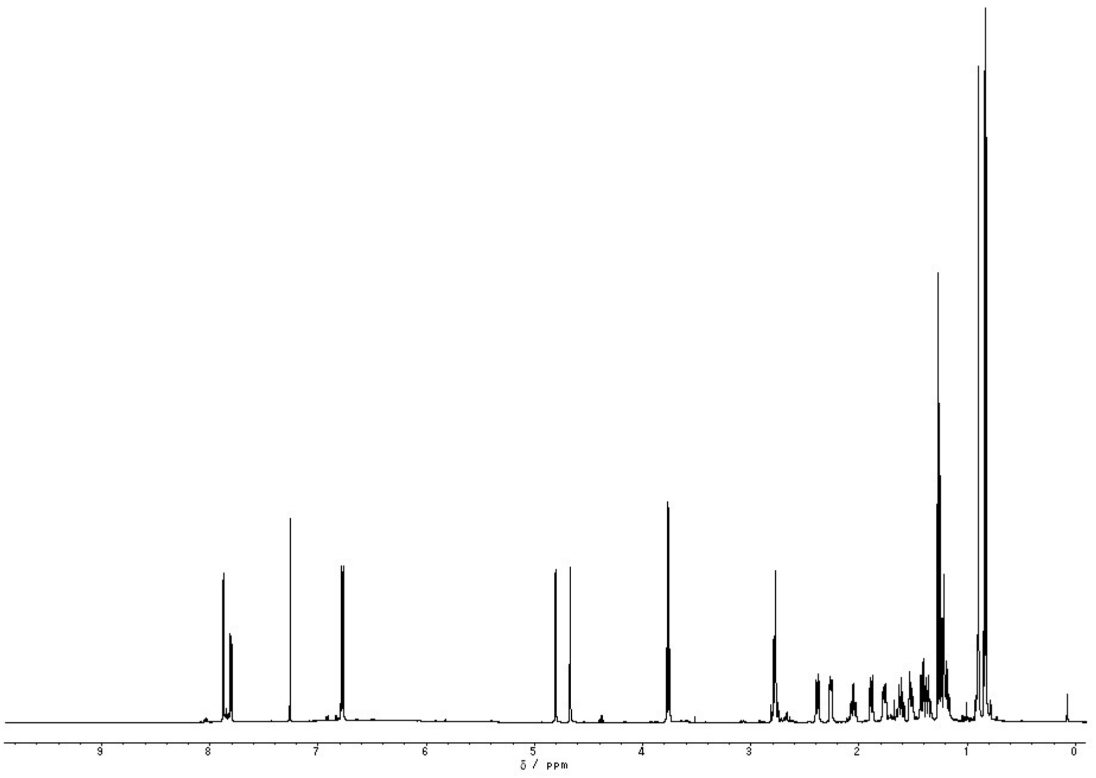

Figure S12: ^{1}H NMR spectrum of zonaroic acid (**4**) in $CDCl_3$

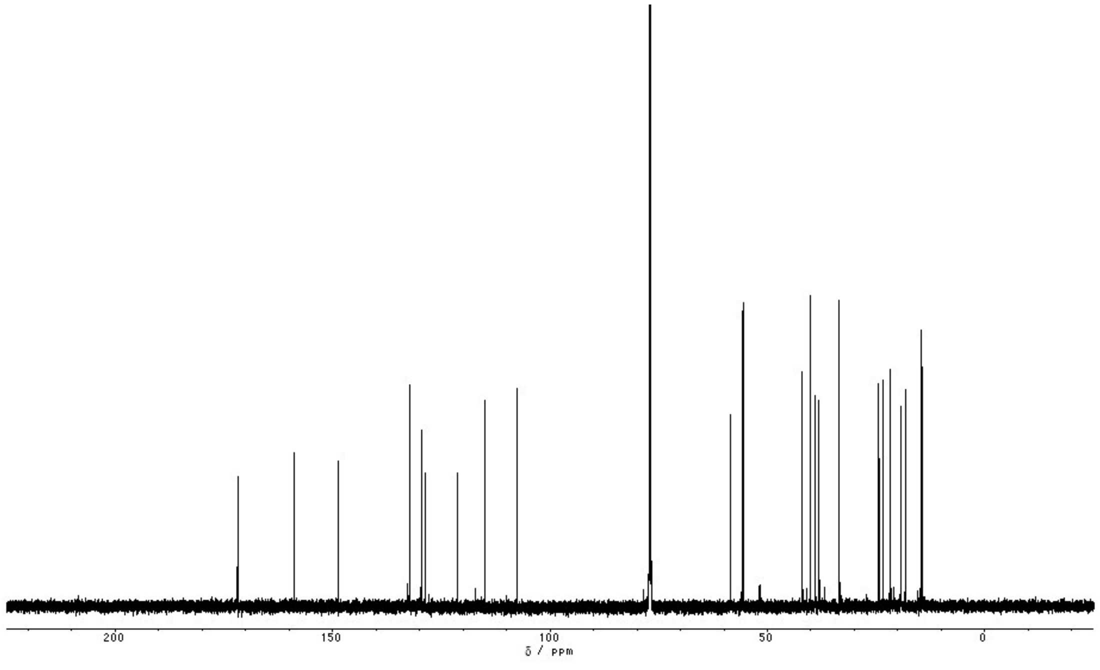

Figure S13: ^{13}C NMR spectrum of zonaroic acid (**4**) in $CDCl_3$

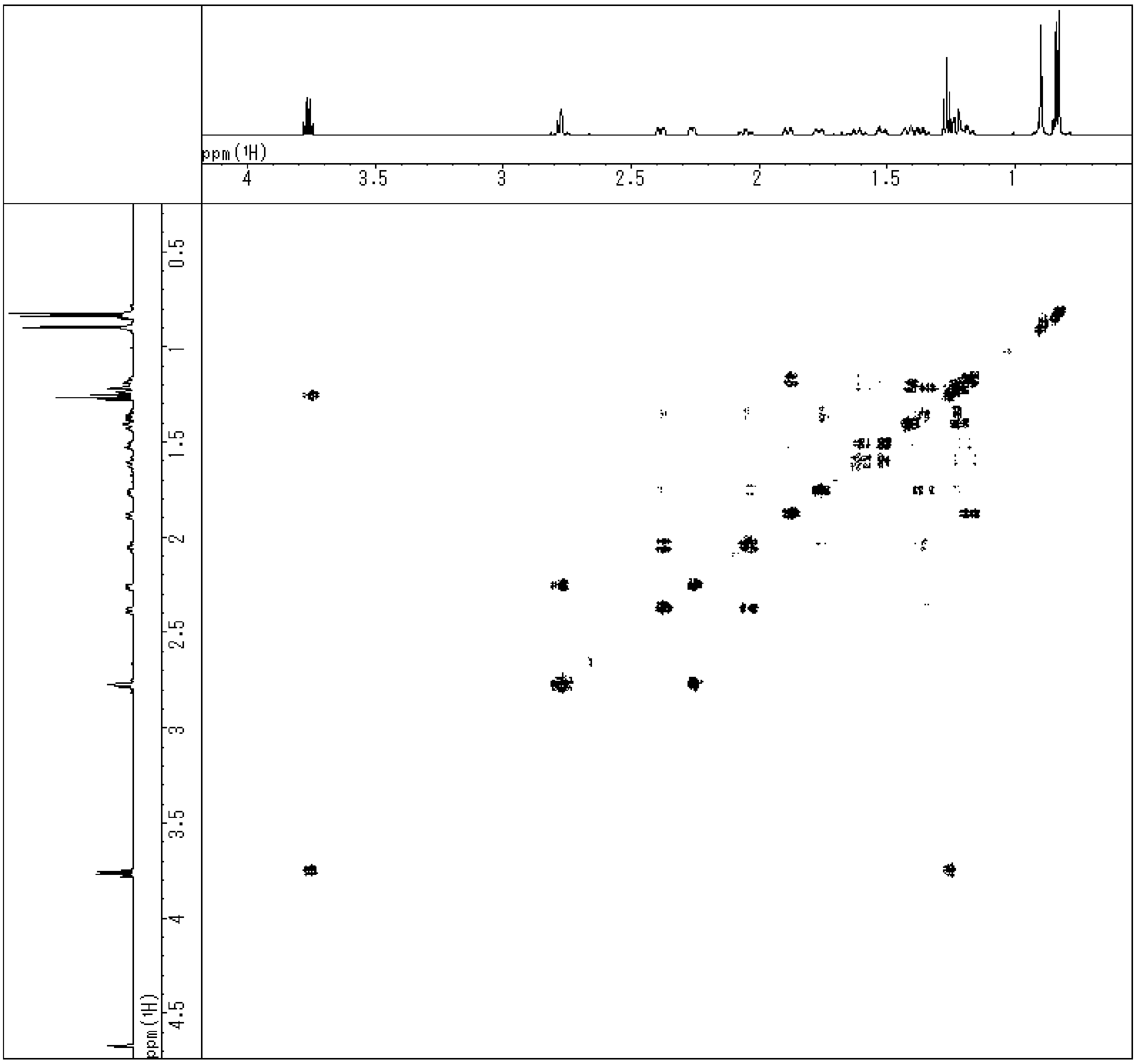

Figure S14: COSY spectrum of zonaroic acid (**4**) in $CDCl_3$

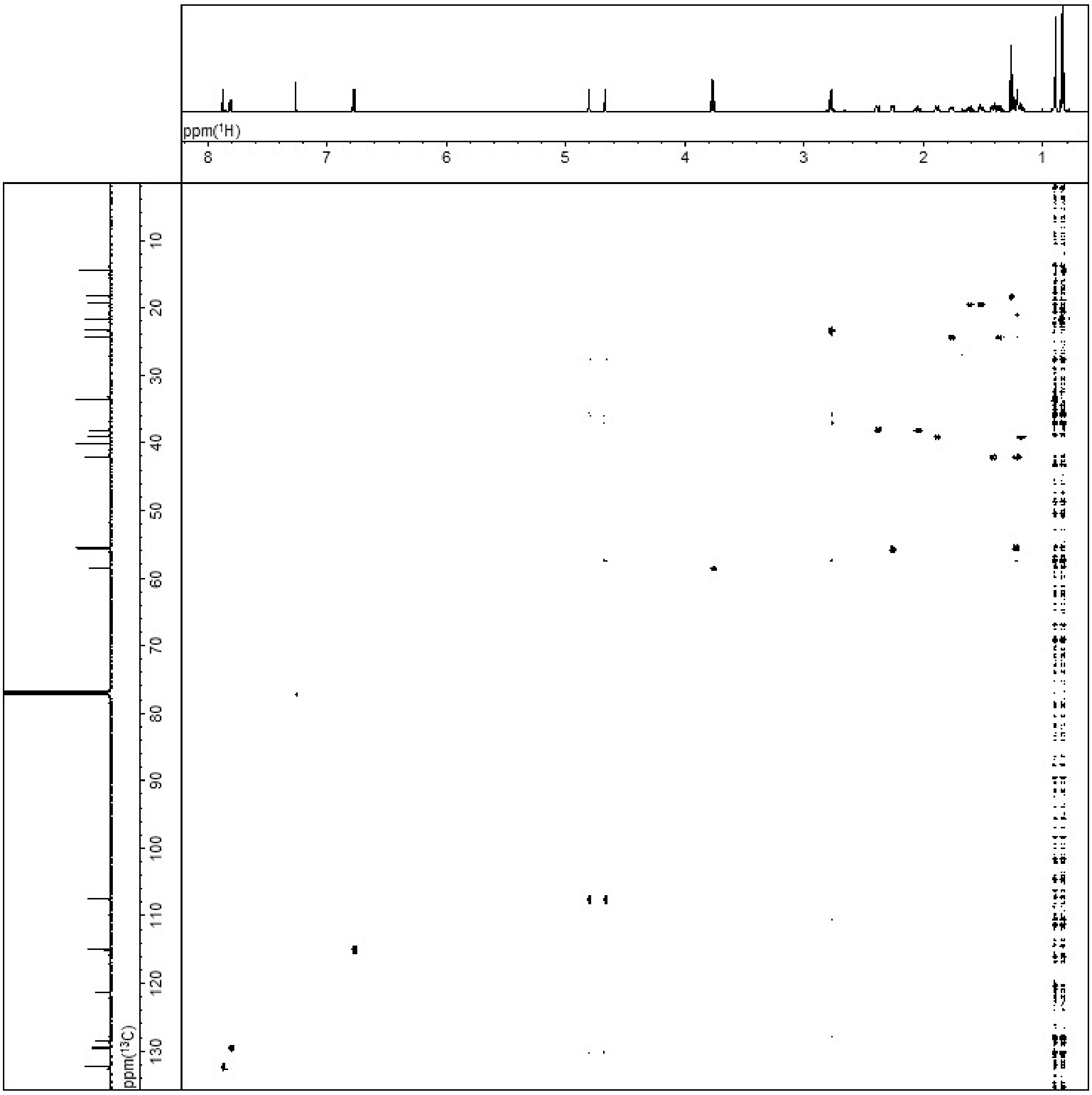

Figure S15: HSQC spectrum of zonaroic acid (**4**) in $CDCl_3$

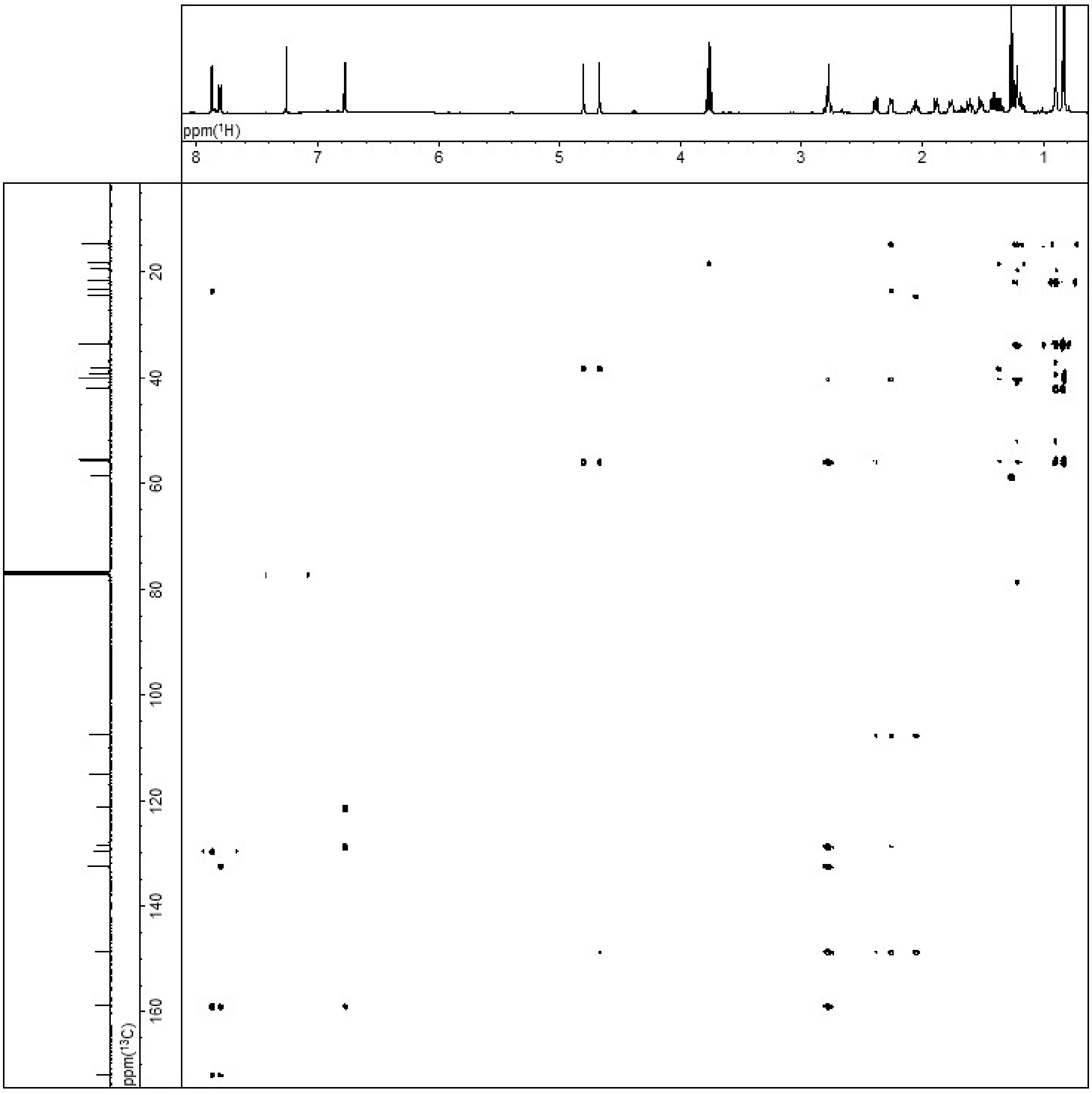

Figure S16: HMBC spectrum of zonaroic acid (**4**) in $CDCl_3$

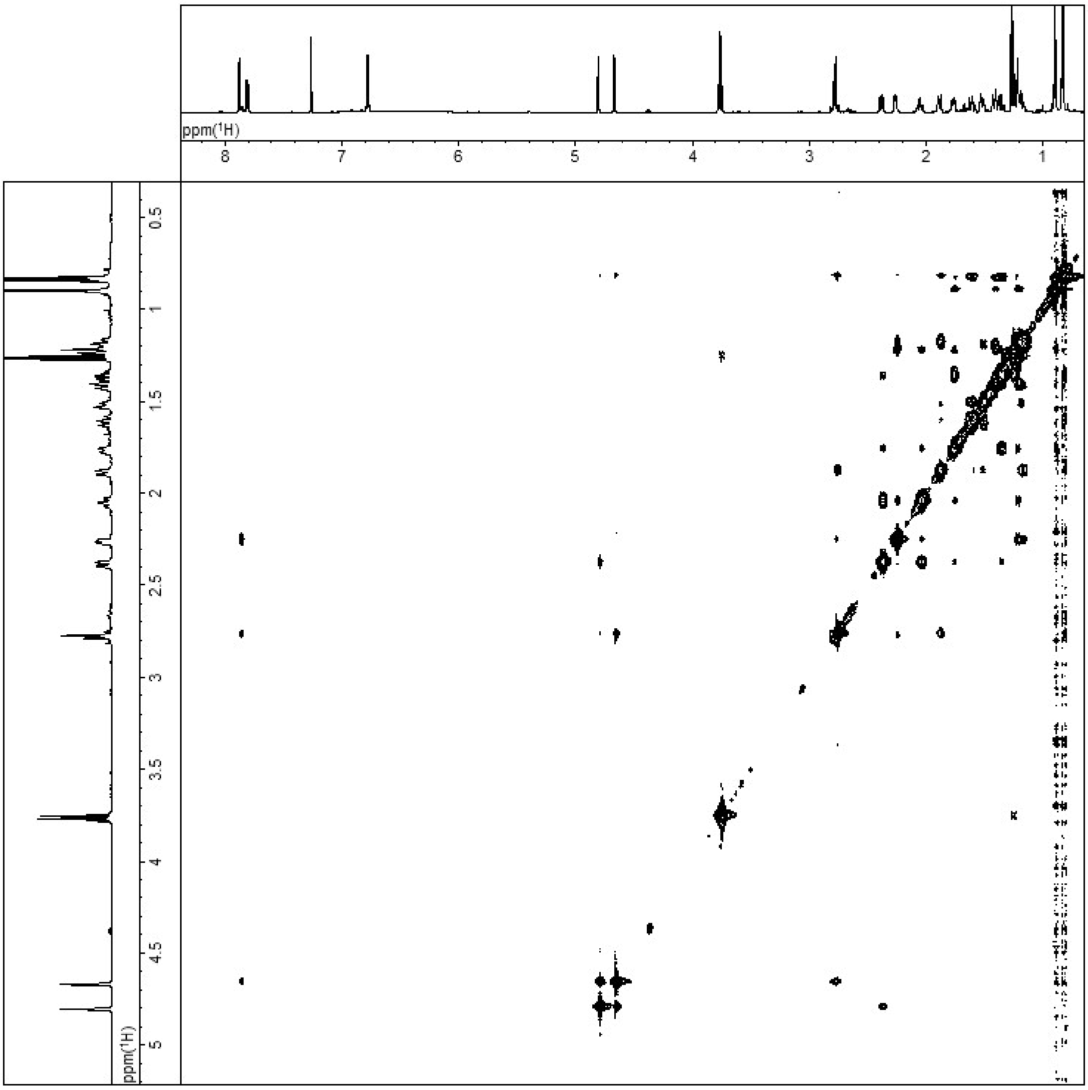

Figure S17: NOESY spectrum of zonaroic acid (**4**) in $CDCl_3$

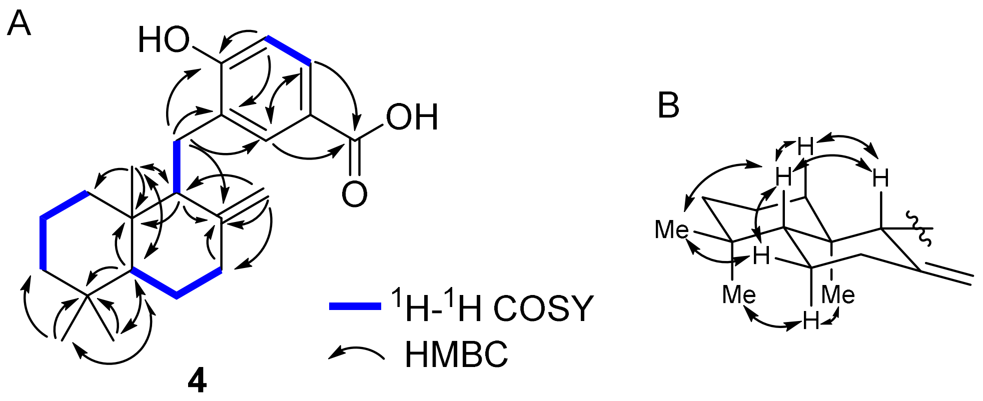

Figure S18: Key COSY, HMBC (A) and NOESY (B) correlations of zonaroic acid (**4**)

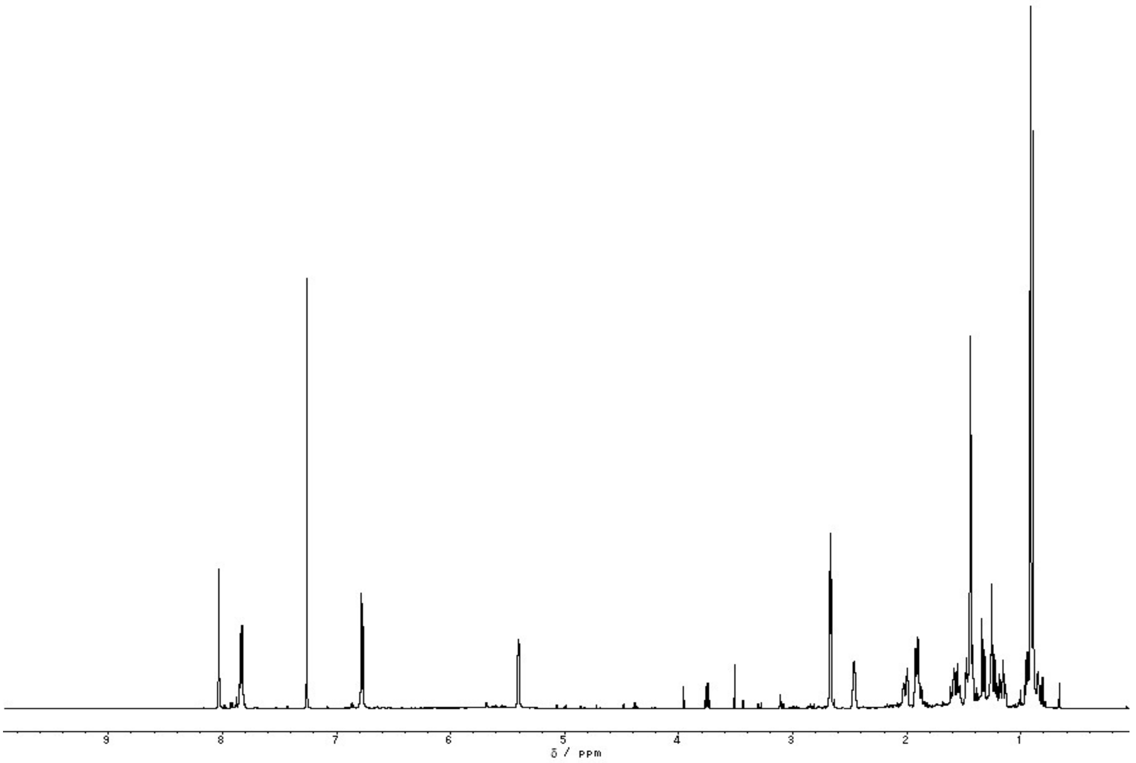

Figure S19: ^{1}H NMR spectrum of isozonaroic acid (**5**) in $CDCl_3$

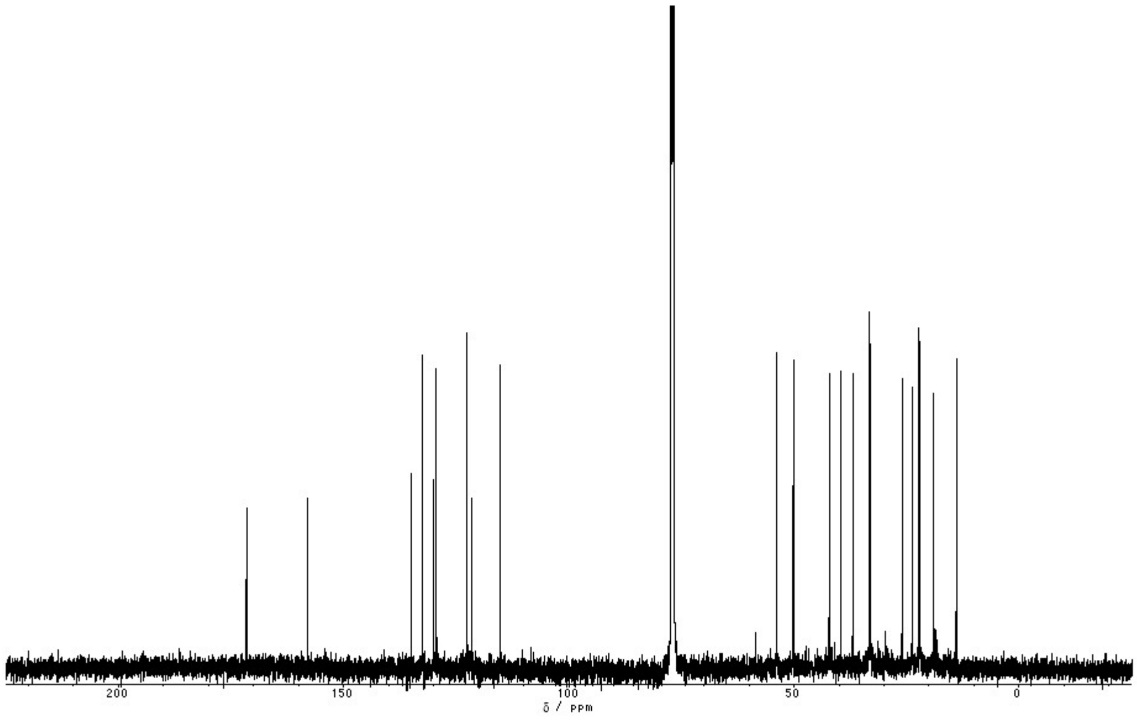

Figure S20: ^{13}C NMR spectrum of isozonaroic acid (**5**) in $CDCl_3$

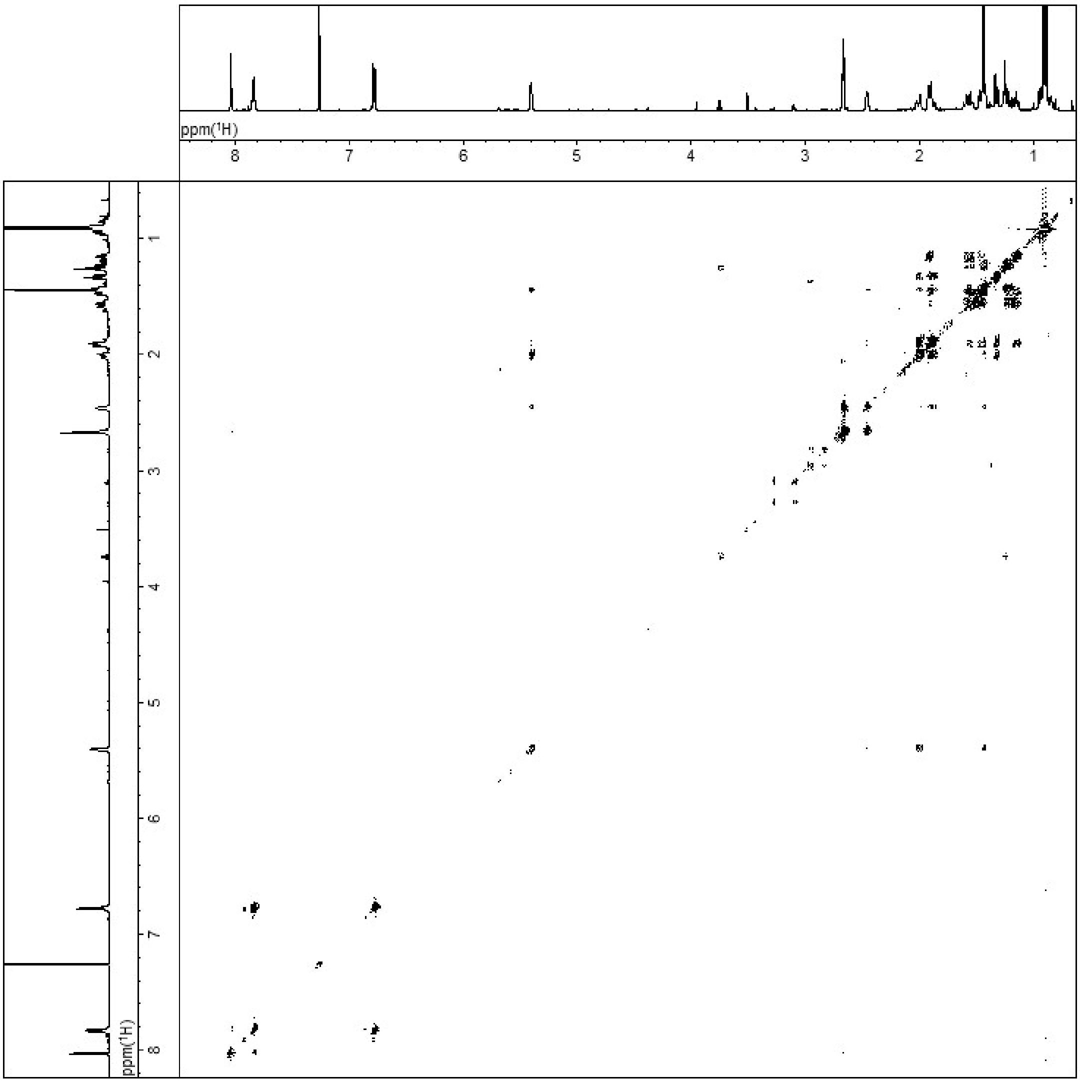

Figure S21: COSY spectrum of isozonaroic acid (**5**) $CDCl_3$

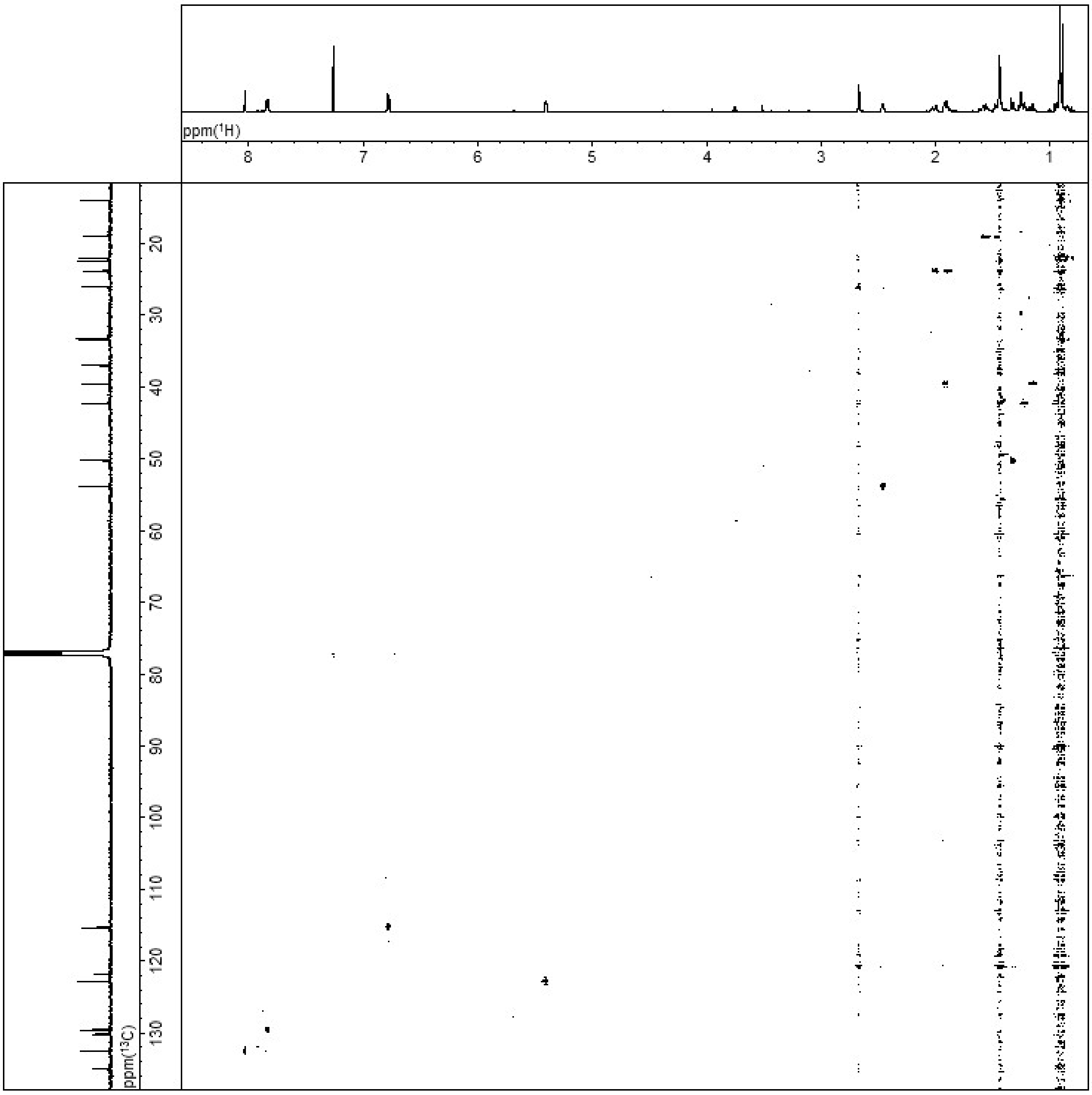

Figure S22: HSQC spectrum of isozonaroic acid (**5**) in $CDCl_3$

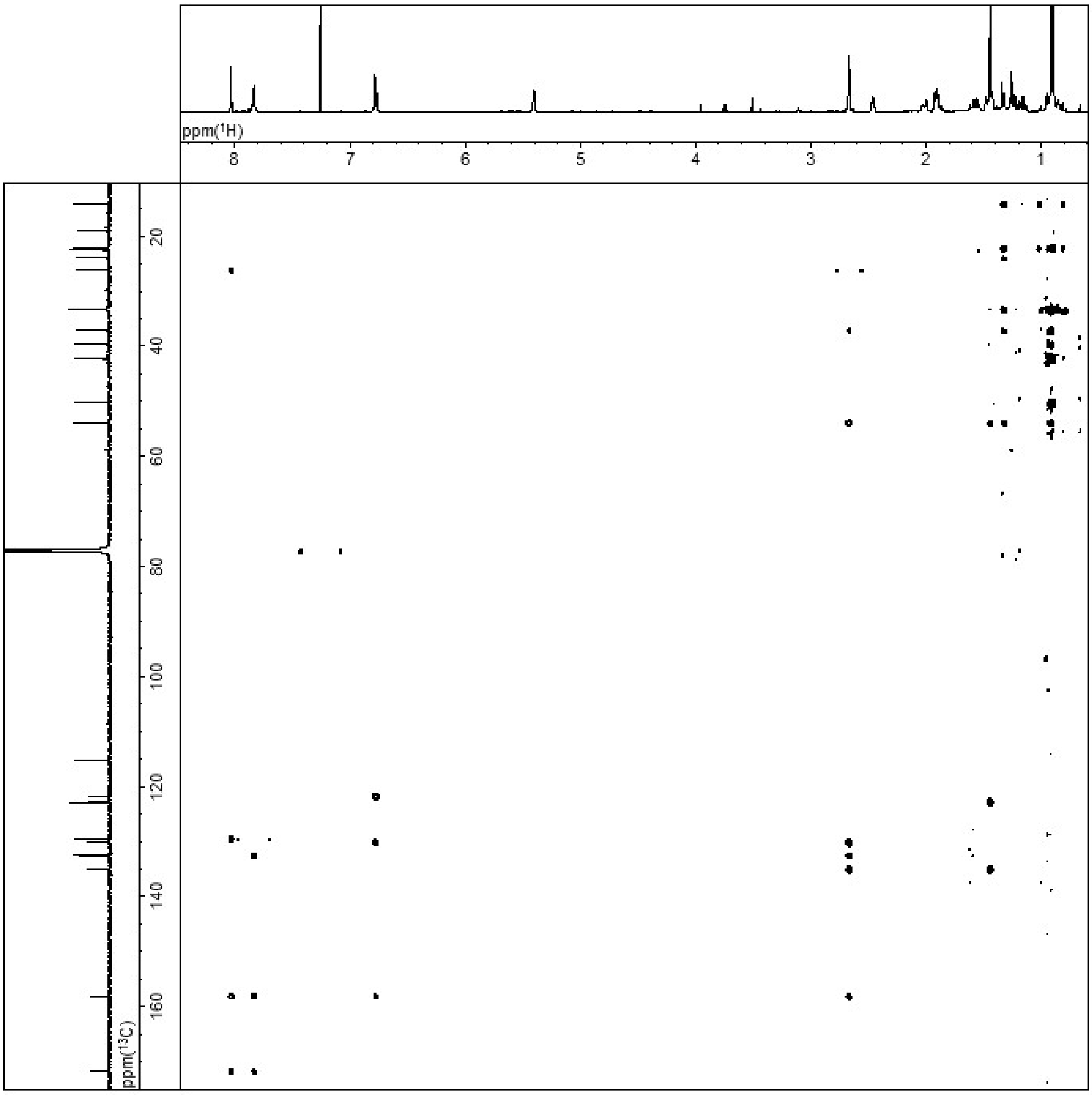

Figure S23: HMBC spectrum of isozonaroic acid (**5**) in $CDCl_3$

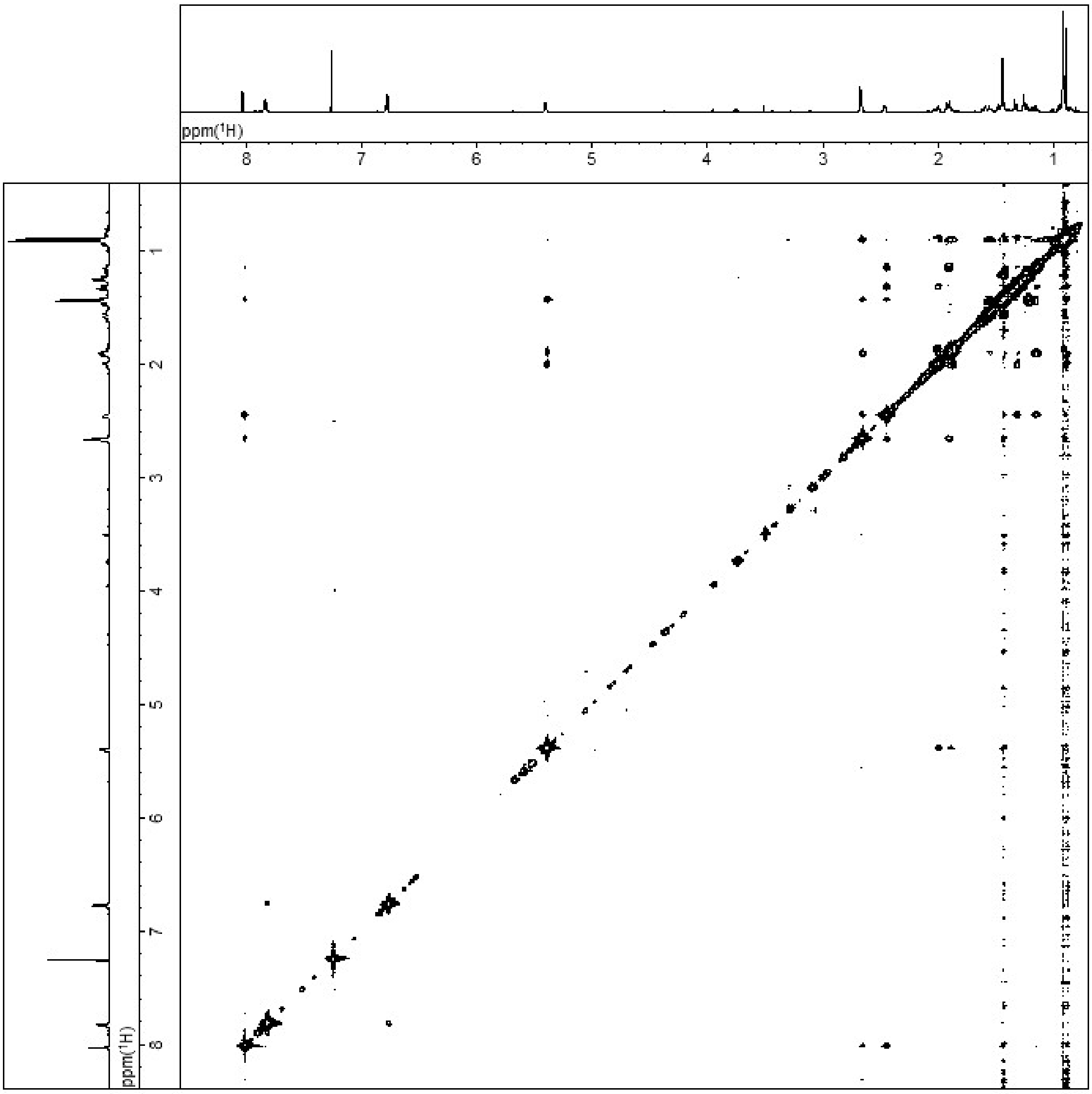

Figure S24: NOESY spectrum of isozonaroic acid (**5**) in $CDCl_3$

Table S1: ^{1}H and ^{13}C NMR spectroscopic data for chromazonarol (**3**), zonaroic acid (**4**) and isozonaroic acid (**5**) in $CDCl_3$

Carbon	Compound **3**		Compound **4**		Compound **5**	
Number	^{13}C	^{1}H	^{13}C	^{1}H	^{13}C	^{1}H
1	39.2	0.95, 1.70	39.1	1.20, 1.88	39.5	1.16, 1.91
2	18.5	1.46, 1.63	19.4	1.61, 1.52	18.9	1.47, 1.57
3	41.8	1.17, 1.40	42.1	1.41, 1.24	42.1	1.23, 1.43
4	33.2		33.6		33.1	
5	56.1	1.02	55.5	1.21	50.1	1.33
6	19.7	1.35, 1.74	24.4	1.36, 1.76	23.8	1.90, 2.00
7	41.1	1.66, 2.03	38.1	2.05, 2.38	122.7	5.41
8	76.7		148.7		135.0	
9	52.1	1.62	55.8	2.26	53.8	2.46
10	36.8		40.1		36.9	
11	22.5	2.56	23.4	2.78	26.0	2.67
12	20.7	1.17	107.6	4.67, 4.81	22.4	1.44
13	21.6	0.84	21.7	0.84	22.0	0.92
14	33.4	0.90	33.6	0.90	33.2	0.89
15	14.8	0.88	14.5	0.83	13.9	0.91
1'	123.3		128.6		130.0	
2'	115.8	6.55	158.8		158.0	
3'	148.5		115.0	6.77	115.2	6.78
4'	114.2	6.56	129.5	7.81	129.4	7.83
5'	117.5	6.62	121.3		121.7	
6'	147.2		132.3	7.87	132.5	8.03
7'			171.9		171.6	
